# Effects of Muscle Fatigue, Creep, and Musculoskeletal Pain on Neuromuscular Responses to Unexpected Perturbation of the Trunk: A Systematic Review

**DOI:** 10.3389/fnhum.2016.00667

**Published:** 2017-01-04

**Authors:** Jacques Abboud, Arnaud Lardon, Frédéric Boivin, Claude Dugas, Martin Descarreaux

**Affiliations:** ^1^Département D'anatomie, Université du Québec à Trois-RivièresTrois-Rivières, QC, Canada; ^2^Pôle Recherche Clinique Chiropratique, Institut Franco-Européen de ChiropraxieIvry-Sur-Seine, France; ^3^Département des Sciences de L'activité Physique, Université du Québec à Trois-RivièresTrois-Rivières, QC, Canada

**Keywords:** electromyography, kinematics, reflex, spinal stability, low back pain, fatigue, ceep

## Abstract

**Introduction:** Trunk neuromuscular responses have been shown to adapt under the influence of muscle fatigue, as well as spinal tissue creep or even with the presence of low back pain (LBP). Despite a large number of studies exploring how these external perturbations affect the spinal stability, characteristics of such adaptations remains unclear.

**Aim:** The purpose of this systematic review was to assess the quality of evidence of studies investigating trunk neuromuscular responses to unexpected trunk perturbation. More specifically, the targeted neuromuscular responses were trunk muscle activity reflex and trunk kinematics under the influence of muscle fatigue, spinal creep, and musculoskeletal pain.

**Methods:** A research of the literature was conducted in Pubmed, Embase, and Sport-Discus databases using terms related to trunk neuromuscular reflex responses, measured by electromyography (baseline activity, reflex latency, and reflex amplitude) and/or trunk kinematic, in context of unexpected external perturbation. Moreover, independent variables must be either trunk muscle fatigue or spinal tissue creep or LBP. All included articles were scored for their electromyography methodology based on the “Surface Electromyography for the Non-Invasive Assessment of Muscles (SENIAM)” and the “International Society of Electrophysiology and Kinesiology (ISEK)” recommendations whereas overall quality of articles was scored using a specific quality checklist modified from the Quality Index. Meta-analysis was performed on reflex latency variable.

**Results:** A final set of 29 articles underwent quality assessments. The mean quality score was 79%. No effect of muscle fatigue on erector spinae reflex latency following an unexpected perturbation, nor any other distinctive effects was found for back muscle fatigue and reflex parameters. As for spinal tissue creep effects, no alteration was found for any of the trunk reflex variables. Finally, the meta-analysis revealed an increased erector spinae reflex latency in patients with chronic LBP in comparison with healthy controls following an unexpected trunk perturbation.

**Conclusion:** The literature provides some evidence with regard to trunk adaptions in a context of spinal instability. However, most of the evidence was inconclusive due to a high methodological heterogeneity between the studies.

## Introduction

Postural balance is constantly challenged, sometimes unexpectedly, by mechanical forces applied in different directions and continuously triggering postural adjustments. In expected conditions, prior to any movement, the central nervous systems triggers muscles activation/deactivation, and then a movement occurs after a short period of a few milliseconds. These pre-planned adjustments are considered anticipatory postural adjustments (Belen'kii et al., 1967; Bouisset and Do, 2008). For instance, trunk postural adjustments can be represented by early muscle activations (Bouisset and Zattara, 1981; Hodges and Richardson, 1997), as well as increases in muscle activity prior to any external perturbation (Lavender et al., 1993; Cresswell et al., 1994; Moseley et al., 2003) that are believed to contribute to spinal stability. On the other hand, when subjected to unexpected external perturbations of the trunk, muscle activation is delayed (Eriksson Crommert and Thorstensson, 2009), leaving the spine with reduced stability for few milliseconds. Spinal stability is defined as the harmonious cohesion between active muscles surrounding the spine, passive spinal tissues, and neuromuscular control (Panjabi, 1992).

When one or more of these spinal stability components are challenged, adaptations in the trunk system occurs in order to maintain a certain performance level in everyday functional motor tasks. Over the past decades, fundamental research efforts have focused on the quantification of these adaptations through the analysis of trunk muscle activity recordings and trunk kinematic data. Challenges to spinal stability have been commonly investigated using muscle fatigue, spinal tissue creep but also by studying patients with low back pain (LBP). Challenging trunk stability using muscle fatigue has been associated with adaptations in muscle activity recruitment patterns, such as trunk flexor and extensor co-contraction phenomena (Allison and Henry, 2001). Reorganization in spatial low back muscle activity have also been described as a potential strategy to offset muscle fatigue effects (Tucker et al., 2009; Abboud et al., 2014). Moreover, the observation of an altered coordination of trunk muscle activation, a decreased control of trunk movements (Boucher et al., 2012), and alterations in lumbopelvic dynamics have been reported under the influence of muscle fatigue (Descarreaux et al., 2010). Similar trunk neuromuscular adaptations have been observed when passive tissue components of spinal stability are challenged. Active or passive prolonged deep flexions of the trunk, which is believed to generate spinal tissue creep, are usually followed by an increase in trunk flexion range of motion (Rogers and Granata, 2006; Howarth et al., 2013; Olson, 2014). Moreover, an increase in back muscle activity has also been described as a compensating mechanism for the reduced contribution of passive tissues to spinal stability (Olson et al., 2004; Shin et al., 2009; Abboud et al., 2016). Neuromuscular control of the trunk, such as trunk coordination and trunk muscle activation, is commonly altered in patients with chronic LBP (Hodges, 2011; Hodges and Tucker, 2011; Abboud et al., 2014). For example, patients with chronic LBP show longer time-delay of trunk muscle activation during a predictable perturbation (Hodges and Richardson, 1998). Overall, challenges to spinal stability components have been associated with numerous alterations in trunk neuromuscular control.

To investigate neuromuscular adaptations to unexpected trunk perturbations, most studies report adaptations in electromyography (EMG) recordings based on the analysis of baseline activity, reflex latency, and reflex amplitude. However, high heterogeneity in EMG reflex variable analyses led to conflicting results and an incomplete understanding of stabilizing responses to unexpected trunk perturbations. Indeed, most of the studies report different criteria to detect spinal reflex parameters. For instance, baseline activity, also called pre-activation level, is calculated from different time windows ranging from 50-ms to 3-s prior to the onset of an unexpected trunk perturbation (Newcomer et al., 2002; Granata et al., 2004, 2005; Herrmann et al., 2006; Rogers and Granata, 2006; Stokes et al., 2006; Mawston et al., 2007; Grondin and Potvin, 2009; Dupeyron et al., 2010; Lariviere et al., 2010; Ramprasad et al., 2010; Bazrgari et al., 2011; Hendershot et al., 2011; Jacobs et al., 2011; Jones et al., 2012a; Liebetrau et al., 2013; Miller et al., 2013; Muslim et al., 2013; Olson, 2014). The onset of EMG reflex, also called reflex latency, is generally calculated, using the standard deviation method proposed by Hodges and Bui (1996). Although, two standard deviations (SD) seems to be the most used reflex onset detection method (Granata et al., 2004, 2005; Herrmann et al., 2006; Rogers and Granata, 2006; Dupeyron et al., 2010; Lariviere et al., 2010; Ramprasad et al., 2010; Bazrgari et al., 2011; Hendershot et al., 2011; Toosizadeh et al., 2013; Olson, 2014), few others studies used alternate standard deviations values, such as 1.4 SD (Radebold et al., 2000, 2001; Cholewicki et al., 2002), 1.5 SD (Reeves et al., 2005), 3 SD (Stokes et al., 2006; Gao et al., 2014; Akbari et al., 2015), and 4 SD (Liebetrau et al., 2013). Sometimes, the EMG reflex onset is also determined by visual inspection of EMG signals (Newcomer et al., 2002; Mawston et al., 2007; Sanchez-Zuriaga et al., 2010). Maximal amplitude value is usually the predominant method to determine reflex amplitude (Granata et al., 2004, 2005; Herrmann et al., 2006; Rogers and Granata, 2006; Grondin and Potvin, 2009; Dupeyron et al., 2010; Sanchez-Zuriaga et al., 2010; Liebetrau et al., 2013; Olson, 2014). However, a few studies have also examined reflex amplitude through EMG time windows of various duration (i.e., 10- to 75-ms windows; MacDonald et al., 2010; Ramprasad et al., 2010; Jacobs et al., 2011; Jones et al., 2012a,b; Shenoy et al., 2013). Finally, and perhaps of utmost importance, authors seems to disagree on what should be consider as reflex responses or voluntary movements. Indeed, some authors consider that muscle activity responses longer than 120-ms should be considered non-reflexive (Granata et al., 2005; Herrmann et al., 2006; Rogers and Granata, 2006; Dupeyron et al., 2010; Jacobs et al., 2011) while other authors included responses occurring between the perturbation onset and 150-ms (Lariviere et al., 2010; Bazrgari et al., 2011; Toosizadeh et al., 2013; Olson, 2014), 200-ms (Liebetrau et al., 2013), 250-ms (Cholewicki et al., 2002), and 300-ms (Radebold et al., 2001).

While there is no doubt that spinal reflexes play a major role in spinal stability mechanisms (Moorhouse and Granata, 2007), well-standardized measurement protocols enabling a better understanding of neurophysiological adaptations to unexpected trunk perturbation are still lacking. Consequently, the main purpose of this study was to systematically assess the quality of evidence of studies investigating neuromuscular responses to unexpected trunk perturbation. More specifically, the targeted neuromuscular responses were trunk muscle activity reflex and trunk kinematics under the influence of muscle fatigue, spinal creep, and musculoskeletal pain. This review also addresses two fundamental questions: What are the most relevant EMG and kinematic variables to properly observe and study neuromuscular adaptions to unexpected loading of the trunk? Are neuromuscular adaptations to unexpected perturbations similar under the influence of erector spinae muscle fatigue, musculoskeletal LBP and spinal creep deformation? We believe that the results of this review will guide future research in the field of trunk neuromuscular control. Moreover, this review may have some potential applications in the development of standardized functional spinal evaluation and biomedical engineering diagnostic tools, as well as progress in ergonomic risk assessment strategies.

## Materials and methods

### Registration

This review protocol was registered in PROSPERO International Prospective Register of Systematic Reviews on May 27, 2016 (CRD42016039374).

### Search strategy

Searches were performed in Pubmed, Embase, and Sport-Discus databases in May 2016 without any time limit. A systematic search of the literature was conducted using the following keywords and search terms alone and in combination: (Perturbation OR Unexpected perturbation OR Postural perturbation OR Sudden release OR Sudden loading OR Quick release OR External load) AND (Back OR Spine OR Spinal OR Trunk OR Lumbar) AND (Muscle fatigue OR Fatigue OR Muscle endurance OR Back pain OR Lumbar impairment OR Stretch OR Creep OR Viscoelastic deformation OR Passive tissue OR Paraspinal tissue OR Prolonged flexion OR Tension–relaxation OR Stiffness OR Static flexion OR Cyclic movement OR Flexion OR Passive movement). Additional data sources included the authors' pre-existing knowledge of the literature, manual review of reference lists of laboratory studies and forward citation tracking. Search strategy is presented in the Appendix A in Supplementary Material.

### Eligibility criteria

Only experimental studies in a controlled environment were selected for this review. Letters, editorials, commentaries, unpublished manuscripts, books and book chapters, conference proceedings, cost analyses, narrative reviews, systematic reviews, clinical practice guidelines were excluded from the study. Studies measuring the effects of any intervention program on trunk stabilization (wearing a lifting belt, rehabilitation, exercise, pharmacology…) were excluded as well. The search strategy was restricted to English and French publications.

Studies were included for subsequent methodological quality assessments if the following criteria were all satisfied: (1) postural perturbation was unexpected; (2) one or more trunk muscle response to postural perturbation was studied; (3) main outcome measure was either trunk muscle reflex recorded with EMG or trunk movement following perturbation; (4) independent variables were erector spinae muscle fatigue or spinal tissue creep or non-specific LBP; (5) human adults participants were tested.

### Study selection

**Figure 1:**
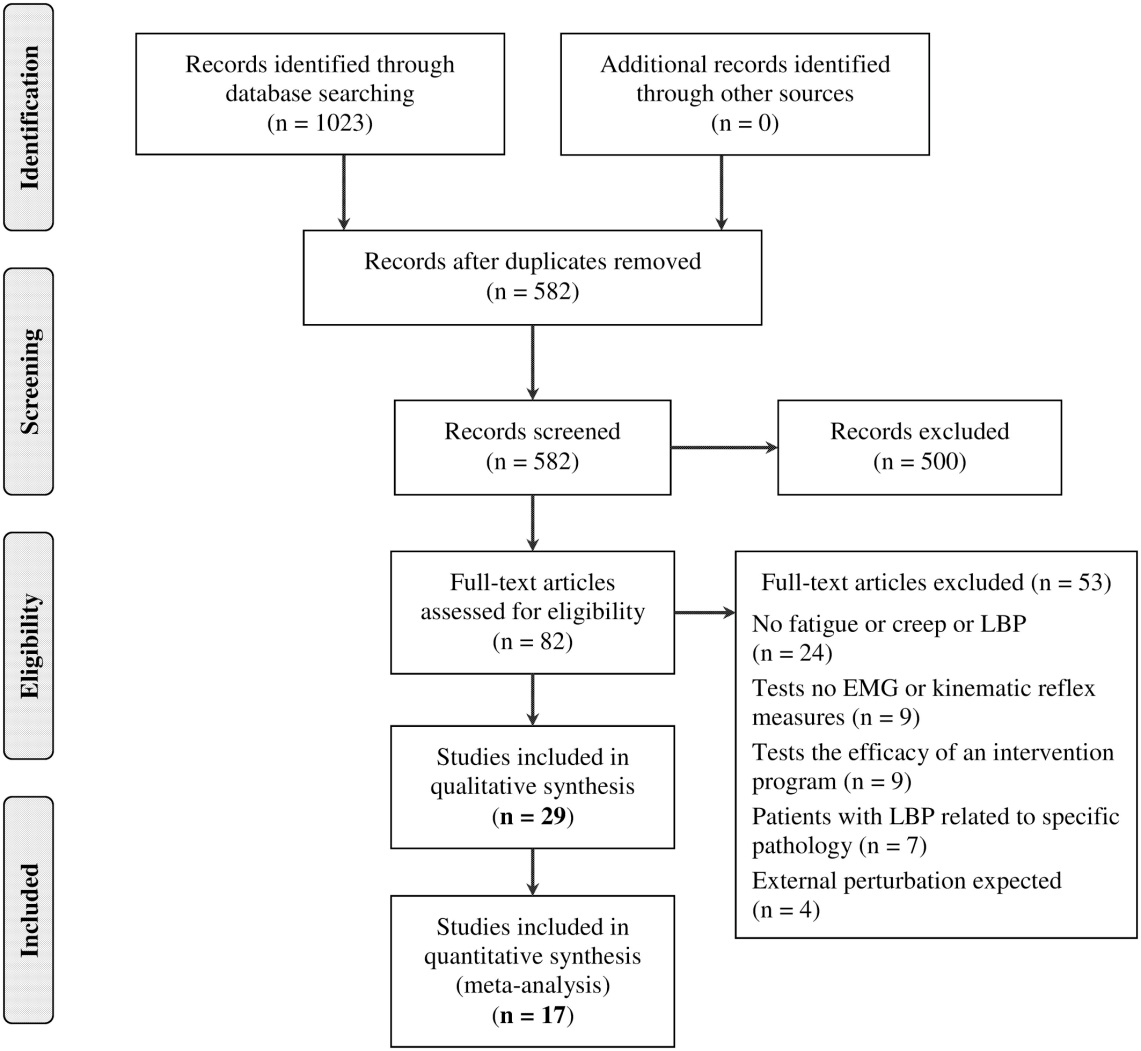
**Preferred Reporting Items for Systematic Reviews and Meta-Analyses (PRISMA) flowchart of the literature search**.

Two independent reviewers (JA, AL) screened citation titles and abstracts to identify the potential eligible articles. A third reviewer (MD) was consulted to resolve any disagreement between the reviewers. Once this first step was done, the relevant full texts were assessed by three independent reviewers (JA, FB, AL) to verify if they could be included in the review according to the five inclusion criteria described previously. In case of disagreement, the two others authors (CD and MD) were consulted. The flowchart of the study has been reported in Figure 1. Excluded articles and the reasons for exclusion were explained in this figure.

### Tools used in the risk of bias assessment

To our knowledge, no validated assessments checklists are available to evaluate the quality of laboratory studies including EMG. Therefore, a custom quality checklist adapted from the Quality Index developed by Downs and Black (Downs and Black, 1998) for the first part and based on the “Surface Electromyography for the Non-Invasive Assessment of Muscles (SENIAM)” (Hermens et al., 1999) and the “International Society of Electrophysiology and Kinesiology (ISEK)” (Merletti, 1999) recommendations for the EMG quality assessment for the second part was created in relation to the specific needs of the objectives of this review.

#### Quality index

The Quality Index is a 27-item checklist for assessing the methodological quality of both randomized and non-randomized studies of health care interventions (Downs and Black, 1998). This tool has been extensively used in the literature and presents a good test-retest reliability (*r* = 0.88) as well as a good inter-rater reliability (*r* = 0.75). From the original 27 items, it was decided during a consensus meeting to create a modified version of the Quality Index using 10 items which were deemed relevant in the assessment of the selected studies (for more details of each items, see Appendix B in Supplementary Material). From the initial Quality Index, 7 items were selected from the category “*reporting part*” (Items 1, 2, 3, 4, 6, 7, 10). Those items assessed if the information provided by the authors allow the reader to have an unbiased view of the study findings. Among the “*external validity”* category, which assesses the overall generalizability of the results, only one item was selected (item 12). Finally, 2 of the 6 items (Items 16, 18) for “*Internal validity—Bias*” were also selected. Each item was scored 0 or 1. When applicable, item 4 “*are the interventions of interest clearly described?*” was divided in two subcategories (item 4.1: description of the perturbation protocol and item 4.2: description of either the muscle fatigue protocol, or the spinal creep protocol). Both of these subcategories were also scored 0 or 1 when applicable. The total maximum score was either 10 or 11 for this section of the checklist.

#### EMG

Electromyography was the main outcome from all the studies included in this review. Based on SENIAM (Hermens et al., 1999) and ISEK (Merletti, 1999) recommendations, the assessment checklist quality was divided in 4 main categories: (1) Surface EMG sensors: 1.1: inter electrode distance, 1.2: material (Ag/AgCl), and 1.3: construction (bipolar). (2) Sensor placement and location: 2.1: Skin preparation, 2.2: placement, and fixation, 2.3: reference electrode and orientation on muscle. (3) Signal processing: 3.1: Filters (type, kind, bandwidth, and order), 3.2: rectification method (full wave, half wave), 3.3: sampling (manufacturer/type of analogue-to digital (A/D) conversion board, sampling frequency, number of bits, input amplitude range), 3.4: amplitude processing (smoothing, average rectified value, root mean square, integrated EMG), and (4) normalization. Each main categories was scored 0 or 1. For the first three main categories, a score of 1 was only attributed when a minimum of 2 items of each category was reported. On the contrary, a score of 0 was attributed. When normalization item was not applicable (i.e., only reflex latency outcome), the total EMG quality score was 3.

#### Quality total score

Depending of the item 4 and normalization scoring, the total maximum quality score was either 13, 14, or 15. The total quality score for each study was expressed as percentage to facilitate comparison between them.

### Risk of bias assessment

Three of the authors (JA, AL, FB) independently assessed the quality of included studies. Assessments were then compared during a formal meeting. Two others authors (CD, MD) were involved in resolving any disagreement between the three first authors when a consensus was not reach after the meeting. If additional information was required to complete the assessment, the corresponding authors of the included studies were contacted.

### Data extraction and synthesis

The first authors (JA) extracted data from the selected studies and completed the evidence table (Appendix C in Supplementary Material).

### Statistical analyses

Inter-rater reliability of the methodological quality checklist was assessed using Fleiss's Kappa statistic with divisions suggested by Landis and Koch (<0.00, poor; 0.00–0.20, slight; 0.21–0.40, fair; 0.41–0.60, moderate; 0.61–0.80, substantial, 0.81–1.00, almost perfect; Landis and Koch, 1977). Meta-analysis could only be performed on reflex latency results because all the authors reported their results with the same unit (ms) while for the other EMG variables, authors reported their results using different units (% of MVC, μV with or without normalization) and it was not possible to pool these outcomes variables together in the same statistical model. To perform the meta-analysis, a random effect model was used since the samples of the included studies did not emanate from the same underlying study population. Meta-analysis were performed using Stata statistical software (College Station, TX: StataCorp LP. 2013). The heterogeneity across the studies was reported as the *I*^2^ (Higgins et al., 2003). It was decided to report a large heterogeneity that was found instead of not reporting the results of the existing evidence.

## Results

### Search results

A total of 582 articles were identified from the literature search and 29 articles fulfilled selection criteria. A summary of the search results is presented in Figure 1.

### Inter-rater reliability

The inter-rater reliability, measured by Kappa values, of all items from the quality checklist and EMG quality checklist ranged from moderate to almost perfect (0.52–1.00). As for the % of disagreement, the highest values were found for checklist items 1 (objectives clearly described) and 2 (EMG reflex outcomes clearly described) with 17% of disagreement, item 4a (perturbation protocol clearly described), 6 (EMG reflex response clearly described), and EMG 2.4 (electrode orientation on muscle) with 14% of disagreement (Table 1). General agreement among raters was at 90% or more for all other methodological quality checklist items. Item 18 (statistical tests appropriate) was excluded from the inter-rater analysis since, an external assessor helped the three authors (JA, AL, FB) assess this item for most of the included studies. A consensus was reached for each article.

**Table 1:** **Inter-rater reliability of quality checklist items [Kappa (95% Confidence interval) and % of disagreement]**.

	**Items**	**Fleiss's Kappa (95% CI)**	**% disagreement**
Quality index checklist	1	0.66 (0.45–0.87)	17
	2	0.52 (0.31–0.73)	17
	3	0.82 (0.61–1.03)	14
	4a	0.58 (0.37–0.79)	7
	4b	1.00	0
	6	0.55 (0.34–0.76)	14
	7	1.00	0
	10	0.85 (0.64–1.06)	10
	12	0.80 (0.59–1.01)	10
	16	1.00	0
EMG quality checklist	1.1	1.00	0
	1.2	0.94 (0.72–1.15)	3
	1.3	0.91 (0.70–1.12)	3
	2.1	1.00	0
	2.2	0.74 (0.53–0.95)	3
	2.3	1.00	0
	2.4	0.78 (0.57–0.99)	14
	3.1	1.00	0
	3.2	0.91 (0.70–1.12)	3
	3.3	1.00	0
	3.4	0.58 (0.37–0.79)	7
	4	0.86 (0.63–1.09)	7

### Quality assessment

Results of the adapted version of the Quality Index are presented in Table 2. The mean score obtained from all the included studies was 75% (ranging from 30 to 100%). Item 3, which relates to the characteristics of participants generally scored poorly. Only 15 studies were considered to provide sufficient information about the inclusion and/or exclusion criteria of their recruited participants. Item 12 relating to the external validity was the one with the lowest score and in only eight studies, authors have identified the source population or recruitment procedure for their participants.

**Table 2:** Quality Index assessment scores (^*^Studies investigated the effect of low back pain were rated using a 10 point scale).

**Authors (year)**	**1**	**2**	**3**	**4a**	**4b**	**6**	**7**	**10**	**12**	**16**	**18**	Score (/10^*^ or /11)	**Score (%)**
Akbari et al., 2015	0	1	1	1	n/a	1	1	1	0	1	0	7^*^	70
Bazrgari et al., 2011	1	0	1	1	1	1	1	1	0	1	1	9	81.8
Dupeyron et al., 2010	1	1	0	1	1	1	1	0	0	1	1	8	72.7
Gao et al., 2014	1	0	1	1	n/a	1	1	1	1	1	1	9^*^	90
Granata et al., 2001	1	0	0	1	1	0	1	0	0	1	1	6	54.6
Granata et al., 2005	1	1	0	1	1	1	1	1	0	1	1	9	81.8
Granata et al., 2004	1	1	0	0	1	1	1	0	0	1	0	6	54.6
Grondin and Potvin, 2009	1	1	0	1	1	1	1	0	0	1	1	8	72.7
Hendershot et al., 2011	1	1	1	1	1	1	1	1	0	1	1	10	90.9
Herrmann et al., 2006	0	1	0	1	1	1	1	1	0	1	1	8	72.7
Jacobs et al., 2011	1	1	1	1	n/a	1	1	1	1	1	1	10^*^	100
Jones et al., 2012a	1	1	1	1	n/a	1	1	0	1	1	0	8^*^	80
Jones et al., 2012b	1	1	1	1	n/a	1	1	1	1	1	1	10^*^	100
Lariviere et al., 2010	1	1	1	1	n/a	1	1	1	0	1	1	9^*^	90
Liebetrau et al., 2013	0	1	0	1	n/a	1	1	0	0	1	1	6^*^	60
MacDonald et al., 2010	1	1	1	1	n/a	1	1	1	0	1	1	9^*^	90
Mawston et al., 2007	1	1	0	1	1	1	1	1	0	1	1	9	81.8
Muslim et al., 2013	1	1	1	1	1	1	1	1	0	1	1	10	90.9
Newcomer et al., 2002	1	1	1	1	n/a	1	1	1	0	1	1	9^*^	90
Olson, 2014	1	1	0	1	1	1	1	1	0	1	1	9	81.8
Radebold et al., 2000	1	1	0	1	n/a	1	1	0	0	1	0	6^*^	60
Radebold et al., 2001	1	1	1	1	n/a	1	1	0	0	1	0	7^*^	70
Ramprasad et al., 2010	1	0	0	1	n/a	0	1	0	1	1	0	5^*^	50
Reeves et al., 2005	0	1	1	1	n/a	1	1	1	0	1	1	8^*^	80
Rogers and Granata, 2006	1	1	0	1	1	1	1	1	0	1	1	9	81.8
Sanchez-Zuriaga et al., 2010	1	1	0	1	1	1	1	1	0	1	1	9	81.8
Shenoy et al., 2013	1	0	0	1	n/a	0	1	0	0	0	0	3^*^	30
Stokes et al., 2006	1	1	0	1	n/a	1	1	0	1	1	0	7^*^	70
Toosizadeh et al., 2013	1	1	1	1	1	1	1	1	0	1	1	10	90.9

As for the EMG quality, the assessment yielded a mean score of 86% (express as percentage, obtained from all the included studies ranging from 50 to 100%; Table 3).

**Table 3:** EMG quality assessment scores (^*^When normalization was not necessary, studies were rated on 3 point scale).

**Authors (year)**	**1.1**	**1.2**	**1.3**	**2.1**	**2.2**	**2.3**	**2.4**	**3.1**	**3.2**	**3.3**	**3.4**	**4**	Score (/3^*^ or /4)
Akbari et al., 2015	1	1	0	1	1	0	1	1	0	1	1	1	4
Bazrgari et al., 2011	0	1	1	0	1	0	0	1	0	1	1	0	2
Dupeyron et al., 2010	0	1	1	1	1	1	1	1	1	1	1	1	4
Gao et al., 2014	0	0	0	1	1	1	1	1	0	1	1	1	3
Granata et al., 2001	0	0	1	1	1	0	1	1	1	1	1	1	3
Granata et al., 2005	0	0	1	0	1	0	0	1	1	1	1	1	2
Granata et al., 2004	0	0	1	0	1	0	1	1	1	1	1	1	3
Grondin and Potvin, 2009	1	1	1	1	1	1	1	1	1	1	0	1	4
Hendershot et al., 2011	0	1	1	1	1	0	0	1	1	1	1	0	3
Herrmann et al., 2006	1	1	0	1	1	0	1	1	1	1	1	1	4
Jacobs et al., 2011	1	1	1	1	1	0	1	1	1	1	1	1	4
Jones et al., 2012a	1	1	1	1	1	0	1	1	1	1	1	1	4
Jones et al., 2012b	1	1	1	1	1	0	1	1	1	1	1	1	4
Lariviere et al., 2010	0	0	0	1	1	1	1	1	1	1	1	1	3
Liebetrau et al., 2013	1	1	1	1	1	1	1	1	1	1	1	0	3
MacDonald et al., 2010	1	1	1	n/a	1	1	1	1	1	1	1	0	3
Mawston et al., 2007	1	1	1	1	1	0	1	1	1	1	1	1	4
Muslim et al., 2013	0	1	1	1	1	0	0	1	0	1	1	0	3
Newcomer et al., 2002	1	0	1	1	1	0	0	1	0	1	1	n/a	3^*^
Olson, 2014	1	1	1	1	1	1	1	1	1	1	1	1	4
Radebold et al., 2000	1	1	1	1	1	0	1	1	1	1	1	n/a	3^*^
Radebold et al., 2001	1	1	1	1	1	0	1	1	1	1	1	n/a	3^*^
Ramprasad et al., 2010	1	1	1	1	1	1	1	1	1	1	1	1	4
Reeves et al., 2005	1	1	1	0	1	0	0	1	1	1	1	n/a	2^*^
Rogers and Granata, 2006	1	1	1	0	1	1	0	1	1	1	1	1	4
Sanchez-Zuriaga et al., 2010	1	1	0	1	1	1	1	1	1	1	1	0	3
Shenoy et al., 2013	1	0	1	1	1	1	1	1	1	1	1	0	3
Stokes et al., 2006	1	1	1	1	1	1	1	1	1	1	1	1	4
Toosizadeh et al., 2013	0	1	1	0	1	0	0	1	1	1	1	1	3

The total quality score, including the Quality Index and EMG, was 79% (ranging from 43 to 100%; Table 4).

**Table 4:** Quality total score (^**#**^Studies investigated the effect of low back pain when normalization was not necessary were rated using a 13 point scale; ^*^Studies investigated the effect of low back pain were rated using a 14 point scale).

**Authors (year)**	Score quality index (/10^*^ or /11)	Score EMG (/3^*^ or /4)	Total score (/13^#^ or /14^*^ or /15)	**Note (%)**
Akbari et al., 2015	7^*^	4	11^*^	78.6
Bazrgari et al., 2011	9	2	11	73.3
Dupeyron et al., 2010	8	4	12	80
Gao et al., 2014	9^*^	3	12^*^	85.7
Granata et al., 2001	6	3	9	60
Granata et al., 2005	9	2	11	73.3
Granata et al., 2004	6	3	9	60
Grondin and Potvin, 2009	8	4	12	80
Hendershot et al., 2011	10	3	13	86.7
Herrmann et al., 2006	8	4	12	80
Jacobs et al., 2011	10^*^	4	14^*^	100
Jones et al., 2012a	8^*^	4	12^*^	85.7
Jones et al., 2012b	10^*^	4	14^*^	100
Lariviere et al., 2010	9^*^	3	12^*^	85.7
Liebetrau et al., 2013	6^*^	3	9^*^	64.3
MacDonald et al., 2010	9^*^	3	12^*^	85.7
Mawston et al., 2007	9	4	13	86.7
Muslim et al., 2013	10	3	13	86.7
Newcomer et al., 2002	9^*^	3^*^	12^#^	92.3
Olson, 2014	9	4	13	86.7
Radebold et al., 2000	6^*^	3^*^	9^#^	69.2
Radebold et al., 2001	7^*^	3^*^	10^#^	76.9
Ramprasad et al., 2010	5^*^	4	9^*^	64.3
Reeves et al., 2005	8^*^	2^*^	10^#^	76.9
Rogers and Granata, 2006	9	4	13	86.7
Sanchez-Zuriaga et al., 2010	9	3	12	80
Shenoy et al., 2013	3^*^	3	6^*^	42.9
Stokes et al., 2006	7^*^	4	11^*^	78.6
Toosizadeh et al., 2013	10	3	13	86.7

### Muscle fatigue

**Figure 2:**
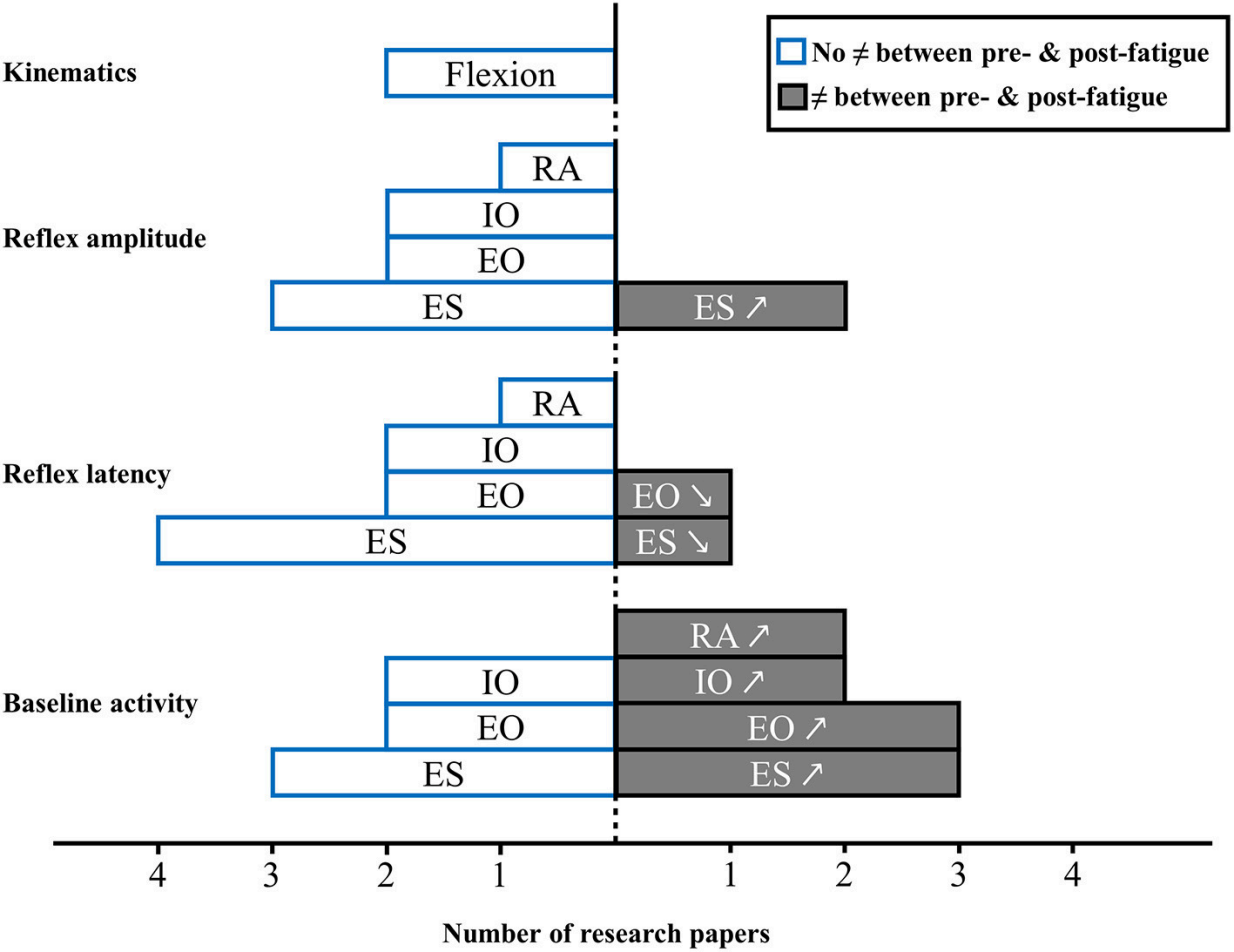
**Muscle activity reflex responses to unexpected postural perturbation of the trunk under the influence of muscle fatigue expressed in number of research papers (↗, higher value with muscle fatigue; ↘, lower value with muscle fatigue; RA, rectus abdominis; IO, internal obliquus; EO, external obliquus; ES, erector spinae)**.

A total of 7 studies investigated the effect of erector spinae muscle fatigue and neuromuscular adaptations following unexpected perturbation of the trunk (Granata et al., 2001, 2004; Herrmann et al., 2006; Mawston et al., 2007; Grondin and Potvin, 2009; Dupeyron et al., 2010; Sanchez-Zuriaga et al., 2010). Figure 2 provides an overview of results drawn from these studies.

#### Baseline activity

Among these studies, results for erector spinae muscle baseline activity prior to an unexpected perturbation was increase after a fatigue task (Granata et al., 2001, 2004; Grondin and Potvin, 2009), while three others studies found no impact on erector spinae baseline activity under the influence of muscle fatigue (Herrmann et al., 2006; Mawston et al., 2007; Dupeyron et al., 2010). As for abdominal muscles, baseline activity results were also mixed. Baseline activity was found to increase after an erector spinae muscle fatigue task for external obliquus in 3 studies (Granata et al., 2001, 2004; Grondin and Potvin, 2009), and for internal obliquus in 2 studies (Granata et al., 2001; Grondin and Potvin, 2009). Conversely 2 other studies found no difference for external obliquus (Mawston et al., 2007; Dupeyron et al., 2010) and internal obliquus muscles (Granata et al., 2004; Mawston et al., 2007). Finally, two studies reported a higher rectus abdominis baseline activity under the influence of erector spinae muscle fatigue (Granata et al., 2001, 2004).

#### Reflex latency

**Figure 3:**
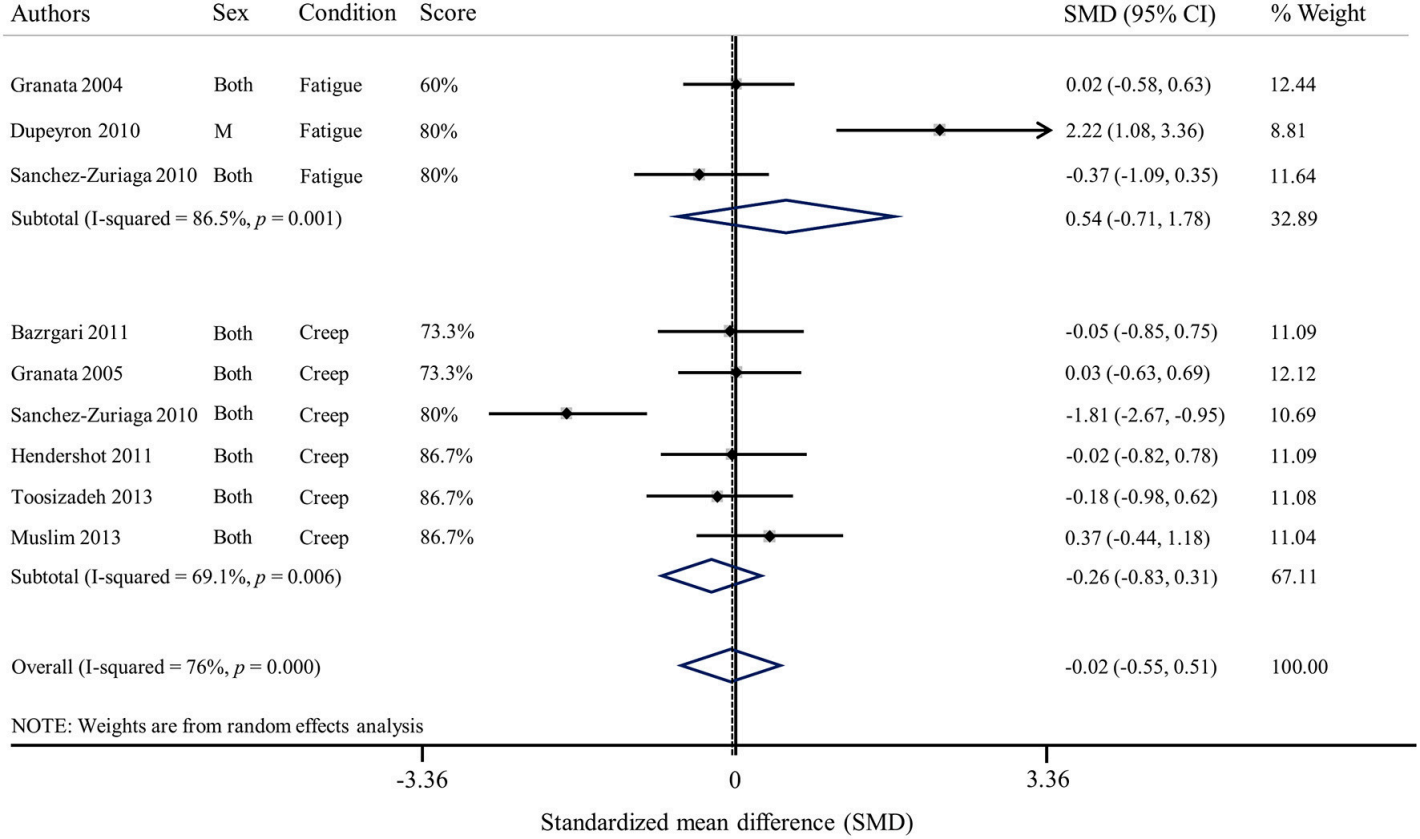
**Forest plot of erector spinae reflex latencies under the influence of muscle fatigue and spinal tissue creep (SMD, Standardized mean difference)**.

In the presence of lower back muscle fatigue, reflex latency of erector spinae muscles was not affected in the majority of studies (Granata et al., 2004; Herrmann et al., 2006; Dupeyron et al., 2010; Sanchez-Zuriaga et al., 2010). One study indicated that the reflex latency was significantly decreased after a fatigue protocol involving erector spinae muscles (Mawston et al., 2007). Following an unexpected perturbation, reflex latency of the internal (Granata et al., 2004; Mawston et al., 2007) and external obliquus (Granata et al., 2004; Dupeyron et al., 2010) and rectus abdominis (Granata et al., 2004) was found to be unchanged in the presence of muscle fatigue. In opposition, one study showed a decrease external obliquus reflex latency after erector spinae muscles fatigue (Mawston et al., 2007). Altogether, results of the meta-analysis shows that there is no effect of muscle fatigue on reflex latency of erector spinae muscles [Standardized mean difference (SMD) = 0.54; 95%CI: −0.71, 1.78; *I*^2^ = 86.5%; Figure 3].

#### Reflex amplitude

In 3 studies, reflex amplitude of the erector spinae was similar with or without muscle fatigue (Granata et al., 2004; Grondin and Potvin, 2009; Sanchez-Zuriaga et al., 2010), while 2 studies found an increased reflex amplitude with fatigue (Herrmann et al., 2006; Dupeyron et al., 2010). External and internal obliquus (Granata et al., 2004; Grondin and Potvin, 2009) and rectus abdominis (Granata et al., 2004) reflex amplitude were not affected by the presence of erector spinae muscle fatigue.

#### Kinematics

Only 2 studies investigated trunk kinematic behavior in response to a sudden perturbation with erector spinae muscle fatigue. These studies did not observe a difference in kinematics between pre and post fatigue condition (Granata et al., 2004; Mawston et al., 2007).

### Spinal creep

**Figure 4:**
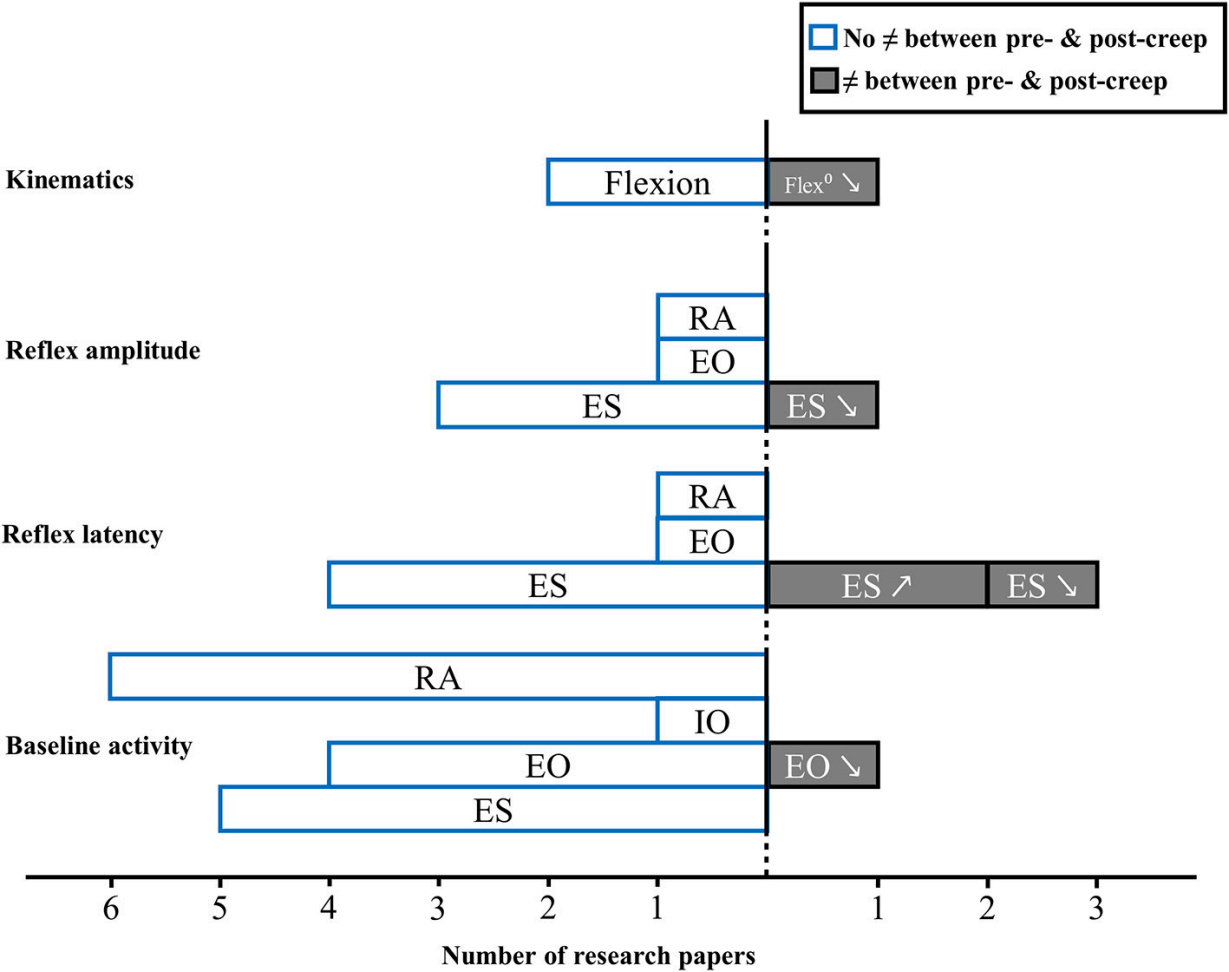
**Muscle activity reflex responses to unexpected postural perturbation of the trunk under the influence of spinal tissue creep expressed in number of research papers (↗, higher value with creep; ↘, lower value with creep; RA, rectus abdominis; IO, internal obliquus; EO, external obliquus; ES, erector spinae)**.

A total of 8 studies reported on the effect of spinal tissue creep and neuromuscular adaptations following unexpected perturbation of the trunk (Granata et al., 2005; Rogers and Granata, 2006; Sanchez-Zuriaga et al., 2010; Bazrgari et al., 2011; Hendershot et al., 2011; Muslim et al., 2013; Toosizadeh et al., 2013; Olson, 2014). Figure 4 provides an overview of results from these studies.

#### Baseline activity

Prior to an unexpected perturbation, baseline activity remained unchanged under the influence of spinal tissue creep for the majority of studies and for all trunk muscles (Granata et al., 2005; Rogers and Granata, 2006; Bazrgari et al., 2011; Hendershot et al., 2011; Muslim et al., 2013; Olson, 2014). Only one study found a decrease in external obliquus baseline activity following creep deformation (Rogers and Granata, 2006).

#### Reflex latency

Two studies showed that, under the influence of spinal tissue creep, reflex latency of the erector spinae muscles increased (Sanchez-Zuriaga et al., 2010; Toosizadeh et al., 2013). Conversely, one study reported that erector spinae reflex latency was shorter in the presence of spinal tissue creep (Muslim et al., 2013). Finally, four studies did not observe significant changes in reflex latency between pre- and post-creep conditions for erector spinae muscles (Granata et al., 2005; Bazrgari et al., 2011; Hendershot et al., 2011; Olson, 2014), as well as for the external obliquus and the rectus abdominis muscles (Olson, 2014). Results of the meta-analysis shows that creep does not have an effect on reflex latency (SMD = −0.26; 95%CI: −0.83, 0.31; *I*^2^ = 69.1%) of erector spinae muscles (Figure 3).

#### Reflex amplitude

Following an unknown perturbation, erector spinae muscle reflex amplitude are generally unaffected by the presence of creep (Granata et al., 2005; Sanchez-Zuriaga et al., 2010; Olson, 2014). One study also found no impact of spinal tissue creep for the external obliquus and the rectus abdominis muscles reflex amplitude following a sudden perturbation (Olson, 2014). Only one study found lower reflex amplitude values following creep deformation for paraspinal muscles (Rogers and Granata, 2006).

#### Kinematics

As for trunk kinematics behavior following an unexpected perturbation under spinal creep condition, two studies found no difference between pre- and post-creep conditions (Rogers and Granata, 2006; Olson, 2014), while one study reported decreased trunk kinematic gain following a creep deformation (Granata et al., 2005).

### Clinical LBP

**Figure 5:**
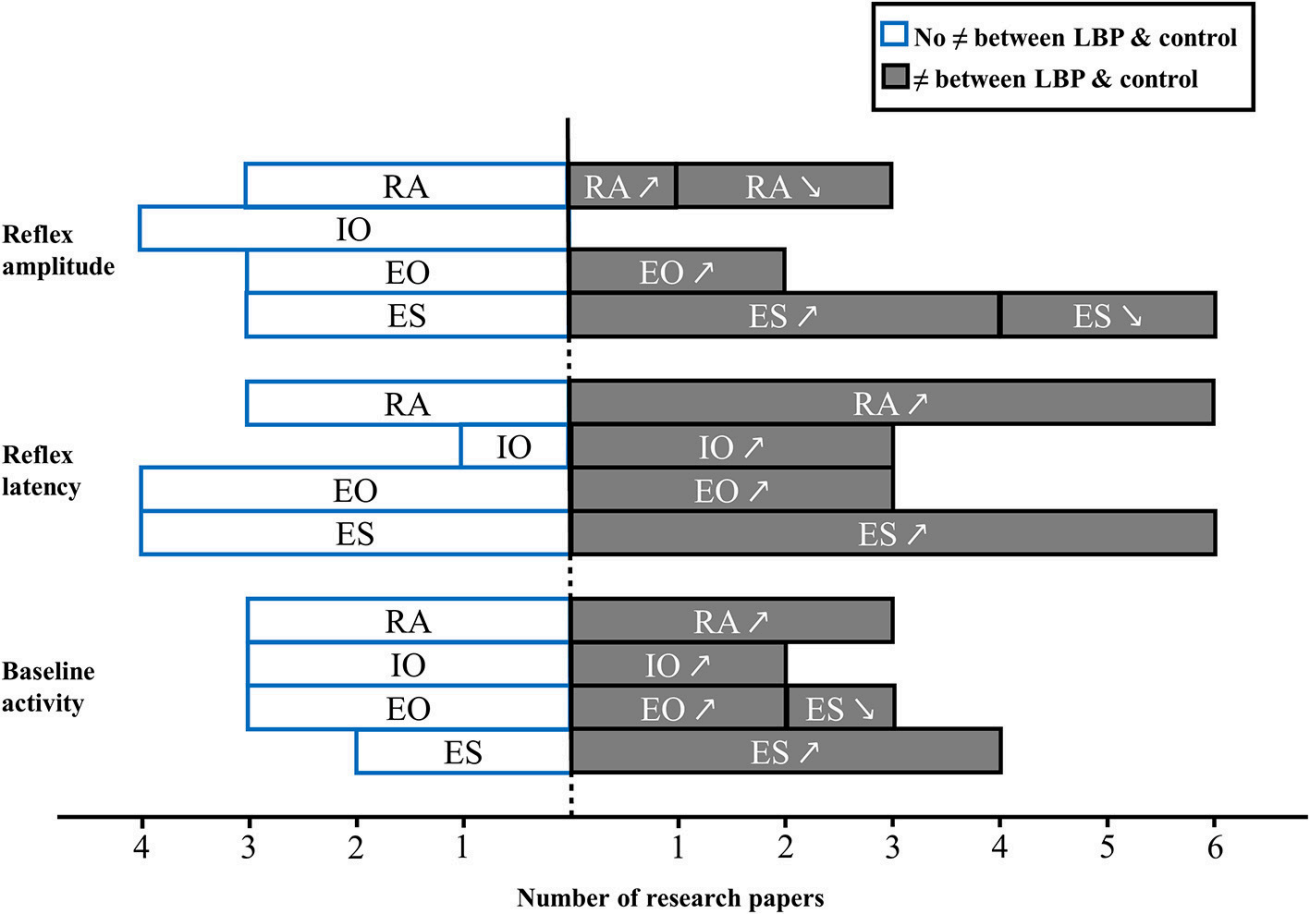
**Muscle activity reflex responses to unexpected postural perturbation of the trunk in patient with LBP expressed in number of research papers (↗, higher value in LBP group; ↘, lower value in LBP group; RA, rectus abdominis; IO, internal obliquus; EO, external obliquus; ES, erector spinae)**.

A total of 15 studies investigated the effect of LBP and neuromuscular adaptations following unexpected trunk perturbation (Radebold et al., 2000, 2001; Newcomer et al., 2002; Reeves et al., 2005; Stokes et al., 2006; Lariviere et al., 2010; MacDonald et al., 2010; Ramprasad et al., 2010; Jacobs et al., 2011; Jones et al., 2012a,b; Liebetrau et al., 2013; Shenoy et al., 2013; Gao et al., 2014; Akbari et al., 2015). Among these studies, two recruited participants with acute/episodic LBP (Stokes et al., 2006; Jones et al., 2012b) while all other included participants with chronic LBP. Figure 5 provides an overview of results drawn from these studies.

#### Baseline activity

Prior to an unexpected perturbation, patients with chronic LBP demonstrated, in most cases, a significant increase of baseline activity of erector spinae muscles (Lariviere et al., 2010; Jacobs et al., 2011; Jones et al., 2012a). An increase in erector spinae baseline activity was also found in patients with acute LBP (Stokes et al., 2006). Nevertheless, three studies failed to identify differences in back muscle baseline activity between healthy participants and patients with chronic LBP (MacDonald et al., 2010; Liebetrau et al., 2013) or acute LBP (Jones et al., 2012b). As for trunk flexor muscles, three different studies did not report any difference between patients with chronic LBP (Lariviere et al., 2010; Liebetrau et al., 2013) or acute LBP (except for the external obliquus baseline activity which decreased in patients; Jones et al., 2012a) and healthy participants, while two studies reported an increase in baseline activity in patients with chronic (Jones et al., 2012a) or acute LBP (Stokes et al., 2006).

#### Reflex latency

**Figure 6:**
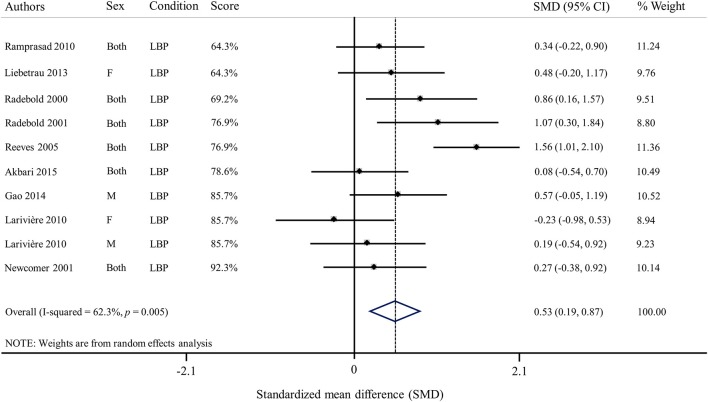
**Forest plot of erector spinae reflex latencies in patients with low back pain (SMD, Standardized mean difference)**.

Among all studies investigating erector spinae reflex latency in patients with LBP, six studies found longer latencies in patients vs. healthy participants (Radebold et al., 2000, 2001; Reeves et al., 2005; Ramprasad et al., 2010; Shenoy et al., 2013; Gao et al., 2014), while four studies did not find significant differences between those two populations (Newcomer et al., 2002; Lariviere et al., 2010; Liebetrau et al., 2013; Akbari et al., 2015). As for external obliquus reflex latencies, results from three different studies showed longer latencies (Radebold et al., 2000, 2001; Reeves et al., 2005), while four studies did not find differences between patients with chronic LBP and healthy participants (Lariviere et al., 2010; Liebetrau et al., 2013; Gao et al., 2014; Akbari et al., 2015). Three studies reported increased internal obliquus reflex latencies in patients with LBP (Radebold et al., 2000, 2001; Liebetrau et al., 2013), while one did not (Akbari et al., 2015). Lastly, patients with LBP exhibited significantly longer reflex latencies over the rectus abdominis muscles in a majority of studies (Radebold et al., 2000, 2001; Reeves et al., 2005; Ramprasad et al., 2010; Liebetrau et al., 2013; Shenoy et al., 2013). However, three studies failed to identify differences between LBP patients and controls (Newcomer et al., 2002; Lariviere et al., 2010; Akbari et al., 2015). Overall, results of the meta-analysis showed that erector spinae reflex latency was increased in patients with LBP vs. healthy participants (SMD = 0.53; 95%CI: 0.19, 0.87; *I*^2^ = 62.3%; Figure 6).

#### Reflex amplitude

Erector spinae reflex amplitude behavior in response to a sudden perturbation seemed in most cases to be increased in patients with chronic LBP (Lariviere et al., 2010; Jones et al., 2012a; Gao et al., 2014) as well as in patients with acute LBP (Jones et al., 2012b). However, two studies reported a decreased erector spinae muscle reflex amplitude in patients with chronic LBP (Ramprasad et al., 2010; Shenoy et al., 2013), while two others studies failed to identify differences between patients with chronic LBP and healthy controls (Jacobs et al., 2011; Liebetrau et al., 2013). Moreover, one study has found that the superficial multifidus reflex amplitude decreased in patients with chronic LBP compared to controls (MacDonald et al., 2010), while one study failed to found a difference between patients with chronic LBP and controls (Liebetrau et al., 2013). As for trunk flexors reflex amplitude, no difference were reported for internal obliquus muscle in people with acute or chronic LBP as compared to asymptomatic participants. External obliquus reflex amplitude was found to be increased in patients with chronic (Jones et al., 2012a) or acute LBP (Jones et al., 2012b), whereas three studies did not observed any difference (Jacobs et al., 2011; Liebetrau et al., 2013; Gao et al., 2014). Finally, no difference were reported for the rectus abdominis reflex amplitude response to a sudden perturbation between healthy participants and patients with chronic (Jacobs et al., 2011; Liebetrau et al., 2013) or acute LBP (Jones et al., 2012b).

## Discussion

To our knowledge, this is the first systematic review that explores the quality of evidence related to muscle activity reflex in response to unexpected trunk perturbation. Since a high heterogeneity was present among the selected studies, our ability to pool data and draw conclusions was limited.

### Quality assessment

The total score of the methodological quality assessment was 79%, with only one study scoring below 50% (Shenoy et al., 2013). Overall, it seems reasonable to suggest that the quality of the included studies was good. However, when quality checklist items are considered individually, some methodological weaknesses emerge. The characteristics of the participants included in the studies were clearly described in only half of the studies. Most studies only mentioned if their participants were healthy participants or patients with LBP without any further clinical details. In most cases, the description of the control group was limited to “healthy” and when a group of LBP was included, the duration and nature of LBP, or any other medical condition were often omitted in inclusion/exclusion criteria. Lack of specific inclusion and exclusion criteria may lead to inherent heterogeneity in patients responses to perturbation as it is known, that patients with acute or chronic LBP can exhibit various neuromuscular adaptations and that such adaptation may be influence by patient's clinical characteristics (Hodges and Tucker, 2011). Another significant methodological weakness identified was external validity. Indeed, only 6/29 studies identified the source population for the recruited participants. This suggest that the overall generalizability of results to the related population is limited. Furthermore, eight studies had small sample size, with fifteen or less participants included. Having such small sample sizes may lead to statistical power issues, which could potentially lead to type II error (Cohen, 1992). Finally, the lack of information concerning the *p*-values was identified in the methodological assessment. The absence of true *p*-value (*p* ≤ 0.05) can lead to the misinterpretation of significant differences and overall interpretation of study results (i.e., *p* = 0.049 vs. *p* = 0.011). Moreover, even if not considered as a criteria of the Quality Index developed by Downs and Black, the 95% confidence interval should also be presented but was reported in only 3 of the included studies.

On the other hand, high methodological quality was found for the descriptions of sudden external perturbation experimental protocols. Moreover, descriptions of the fatigue and/or creep protocols were also appropriately detailed. This indicates that these experimental protocols would be replicable. Unfortunately, a high heterogeneity between experimental protocols makes the interpretation of the original study results difficult. Indeed, sudden external perturbations were applied in different positions (standing and semi-sitting) with different magnitudes, and sometimes using a familiarization perturbation protocol. As for EMG assessment, the overall quality was good. However, three items drawn from ISEK and SENIAM recommendations were absent in most of the studies: description of the inter electrode distance, the reference electrode and the normalization procedures. The inter electrode distance could influence data recording, due crosstalk effects (Hermens et al., 1999; De Luca et al., 2012), whereas the absence of normalization may lead to misinterpretation of results when comparing the amplitude of muscle activity (reflex amplitude or baseline activity prior to perturbation) between participants (Merletti, 1999).

### Baseline activity

Experiencing an unexpected perturbation limits the nervous system capacity to anticipate and preprogram a motor response. Yet, baseline muscle activity, was one of the most reported variable in studies that evaluated the effect of unexpected perturbation (19/29 of the included studies). Despite the absence of feedforward strategies, small changes in baseline activity have been described under muscle fatigue or in the presence of LBP, while baseline activity is not modified under the influence of spinal creep. Anxiety can also affect postural stability (Wada et al., 2001; Stambolieva and Angov, 2010) and therefore potentially modulate baseline activity while “waiting” for an external perturbation to happen. However, there is not enough evidence to strongly propose that baseline activity can be influenced by the varying perturbation delays. Indeed, this review could not reach any definite conclusion with regard to baseline activity since the included articles did not report specific perturbation delays. Most articles reported a variation of time delay (i.e., between 1 and 10 s) or they did not report any details.

### Muscle fatigue effects on spinal stability

The relationship between muscle fatigue and spinal stability remains unclear. Despite the varying reflex latency values between studies, this review suggests that trunk muscle reflex response latencies do not change under the influence of back muscles fatigue. This suggest that, in order to stabilize the spine, the central nervous system generates earlier postural muscle adjustments similarly regardless of muscle fatigue presence. The results from the metanalysis should, however, be interpreted with caution. An I square superior to 80% suggests the presence of a substantial heterogeneity between those studies. As for baseline activity and reflex amplitude of erector spinae muscles, surprisingly, no consensus was found in this review. Since the presence muscle fatigue is usually characterized by an increase in the EMG amplitude signal in submaximal muscle contractions (De Luca, 1997), a higher trunk muscle EMG amplitude was expected, especially in muscles targeted by the fatigue protocol. The flexor muscle baseline activity and reflex amplitude did not seem to be affected by the presence of back muscle fatigue in most studies and only the rectus abdominis baseline activity increased prior to an unknown perturbation in the presence of muscle fatigue. However, these results should be interpreted with caution since only two studies reported such responses to muscle fatigue. This note of caution can also apply to trunk kinematic behaviors since the lack of any effect of erector spinae muscle fatigue on trunk kinematics was reported in very few studies. Overall, it could be hypothesized that muscle fatigue has a negligible impact on spinal stability. A previous study showed that even in the presence of upper limb muscle fatigue, movement accuracy with external perturbation remains constant (Takahashi et al., 2006). Moreover, the lack of trunk movement changes in a fatiguing state could be explained by the trunk muscle system's redundancy which offers various adaptation possibilities to achieve a similar goal (Latash and Anson, 2006). Investigating neuromuscular strategies such as variability in muscle activity recruitment pattern should shed some light on the effects of trunk muscle fatigue during unexpected trunk perturbations.

### Spinal tissue creep effects on spinal stability

Overall, the presence of spinal tissue creep does not seem to affect spinal stability in a context of unexpected perturbation. Indeed, this review revealed that trunk muscle baseline activity prior to a perturbation does not change following either an active or a static deformation of passive spinal tissues. Following an unknown perturbation, participants showed similar trunk muscle reflex amplitude. Again, active vs. passive deformation do not yield distinct effects on reflex amplitude. Such result is surprising since it is expected that creep deformation will lead to an increase muscle activity amplitude (Olson et al., 2009; Abboud et al., 2016), which is believed to act as a spinal stabilization mechanism. Interesting new findings have shown that following a prolonged intermittent trunk flexion of 1 h, an increase of trunk stiffness is observed (Voglar et al., 2016). This observation confirms previous findings suggesting that in the first 30 min of cyclic trunk flexion, a decrease in intrinsic stiffness occurs, whereas, the following 30 min, spinal stiffness increases (Parkinson et al., 2004). Since spinal stiffness has been associated with spinal stability (Graham and Brown, 2012), it can be hypothesized that no adjustment of reflex amplitude is needed when intrinsic stiffness increases. However, studies included in this review cannot support this hypothesis, since spinal creep deformation lasting from 15 min to 1 h did not modify the reflex amplitude. As for reflex latency, no distinct effect of spinal tissue creep could be identified in the meta-analysis. In most cases, reflex latency did not change in the presence of spinal tissue creep. Once again, the results drawn from the metanalysis should be interpreted with caution due to the heterogeneity between the included studies (*I*^2^ = 69%). Overall, this review suggest that spinal tissue creep had no or only minor effects on trunk neuromuscular adaptations to unexpected perturbation. Moreover, no definite conclusion can be drawn for trunk kinematics since only three studies investigated the effect of spinal tissue creep and reported conflicting results. It seems reasonable to suggest that, in a context of spinal instability, the impact of transient spinal tissue deformation can be counteracted by recruiting other muscle groups and using alternate neuromuscular strategies. Indeed, it has already been proposed that the loss of viscoelastic tissues of ligaments, discs, and joint capsules properties can be counteracted by adjusting the co-contraction levels of agonist and antagonist muscles (Solomonow et al., 1999).

### Musculoskeletal LBP effects on spinal stability

The effect of LBP on spinal stability was the most common topic identified in the current review. Despite the number of studies available, no definite conclusion could be drawn. Results for most EMG reflex variables included in this review were found to be conflicting across studies. More studies found differences between a healthy population and populations of patients with chronic LBP than studies that did not, especially for the trunk muscles baseline activity. Similar observations were found in patients with acute or episodic LBP. On the other hand, studies investigating the effects of acute clinical LBP induced by experimental LBP, consistently reported no change in trunk muscle baseline activity prior to an unexpected perturbation (Gregory et al., 2008; Boudreau et al., 2011; Miller et al., 2013). Despite the overall conflicting observations, results from the meta-analysis showed a moderate effect indicating a longer reflex latency for erector spinae muscles in patients with chronic LBP compared to healthy participants. However, the meta-analysis results for reflex latency should be interpreted with care since the analysis was conducted using reflex latency values that were drawn directly from the article or provided by the authors. Although, no meta-analysis was conducted for reflex amplitude, erector spinae muscle reflex amplitude was found to be significantly higher in patients with acute or chronic LBP in most studies while two studies reported a decrease in the same population. It should be noted that, these two latter studies reporting a lower erector spinae reflex amplitude in patients with chronic LBP were among the studies with the lowest quality score (see Table 4). It is known that patients with LBP are highly heterogeneous and many studies have attempted to identify subgroups (O'sullivan, 2005; Fersum et al., 2010). Patients described in the included studies differed, from a study to another, with regard to their respective pain scores (2–4.7/10 on numerical pain scale), their disability scores (very low to moderate), as well as in the pain duration (3 months to several years). If these subgroups exist, one typical neuromuscular response could be associated with one typical subgroup. The heterogeneity of the results reported in this review highlights the importance of standardized and well described inclusion and exclusion criteria in experimental studies investigating patients' populations.

### Limitations

Since no validated assessment checklist was available to evaluate the quality of laboratory studies using EMG assessments, a custom made quality checklist was adapted from an already validated existing checklist (Downs and Black, 1998). However, to improve the validity and reliability of our checklist, three independent assessors completed the quality checklist and showed a high level of agreement. A methodological limitation of this review is that only one author have extracted the data (Appendix C in Supplementary Material). Another limitation of this review is the limited number of studies investigating muscle fatigue, spinal tissue creep, and musculoskeletal pain effects. Diverse sensorimotor and biomechanical external perturbation, such as vibration (Arashanapalli and Wilson, 2008; Santos et al., 2008; Arora and Grenier, 2013; MacIntyre and Cort, 2014) and delayed onset muscle soreness (Hjortskov et al., 2005), were identified during the preliminary search. However, an insufficient number of studies was available to consider these topics in the review.

This systematic review assessed the overall quality of the included articles. However, due to the large number of included studies, it was not feasible to contact all the authors of articles who omitted methodological details, such as inclusion/exclusion recruitment criterion, electrode placement, etc. Moreover, in order to guide future research, this review was designed to highlight the lack of standardization and information characterizing this type of study. This review did not assess the reliability and validity of the main outcomes. Besides, almost all studies did not provide data about EMG reflex variable reliability and/or validity, we decided not to penalize study who did not report theses information since EMG was already proven to be valid and reliable assessment tool in many studies. Instead, it was chosen to focus on the quality of EMG data acquisition and analyses which are considered key factors in the value and interpretation of results (De Luca, 1997). A final limitation of this review was the incapacity to conduct meta-analysis on variables other than reflex latency. Unfortunately, the use of different units (% of MVC, μV, normalized EMG with no unit, etc.) to express reflex amplitude or baseline activity made the meta-analysis virtually impossible.

### Research recommendations

It is clear that standardization for conducting and reporting EMG fundamental studies should be a priority in future research. The development of an adapted checklist for EMG fundamental and clinical studies may be a helpful tool to achieve such a goal. Moreover, future studies should establish the reliability of the EMG reflex variables. Despite the presence of a good reliability in the determination of reflex latency using SD methods (Hodges and Bui, 1996), reliability or validity of reflex amplitude and baseline activity have not been assessed in most studies. Given the various reflex outcomes studied and the overall heterogeneity of the studies included in this systematic review, determining how physical and physiological reflex responses adapt in various spinal instability conditions should remain an active domain of research. Future research should also consider exploring the impact of spinal instability on trunk kinematic behavior in the presence of expected and unexpected external perturbations.

## Author contributions

Substantial contributions to the conception or design of the work; or analysis, or interpretation of data for the work: JA, AL, FB, CD, MD. Drafting the work and revising it critically for important intellectual content: JA, AL, FB, CD, MD. Final approval of the version to be published: JA, AL, FB, CD, MD. Agreement to be accountable for all aspects of the work in ensuring that questions related to the accuracy or integrity of any part of the work are appropriately investigated and resolved: JA, AL, FB, CD, MD.

## Funding

This study was funded through the Natural Sciences and Engineering Research Council of Canada in the form of both a scholarship and discovery grant.

### Conflict of interest statement

The authors declare that the research was conducted in the absence of any commercial or financial relationships that could be construed as a potential conflict of interest.
