# Supplementary material for: Effects of Muscle Fatigue, Creep, and Musculoskeletal Pain on Neuromuscular Responses to Unexpected Perturbation of the Trunk: A Systematic Review
Source: Front Hum Neurosci. 2017 Jan 4;10:667. doi: 10.3389/fnhum.2016.00667 (PMC5209383; doi:10.3389/fnhum.2016.00667)
Supplement: Supplementary file 1 [file DataSheet1.pdf]

Appendix A. Search strategy

Keywords	Search terms
"Perturbation"	Perturbation
	Unexpected perturbation
	Postural perturbation
	Sudden release
	Sudden loading
	Quick release
	External load
AND	
"Trunk"	Trunk
	Back
	Spine
	Spinal
	Lumbar
AND one of the following keywords	
"Pain"	Pain
	Impairment
	Disorder
OR	
"Creep"	Creep
	Stretch
	Viscoelastic deformation
	Passive tissue
	Paraspinal tissue
	Prolonged flexion
	Tension–relaxation
	Stiffness
	Static flexion
	Cyclic movement
	Flexion
	Passive movement
OR	
"Fatigue"	Muscle fatigue
	Fatigue
	Muscle endurance

Appendix B

Item 1. Is the hypothesis/aim/objective of the study clearly described?
Yes = 1 / No = 0
When hypothesis was mentioned, a score of 1 was only attributed if the authors have stated a bi or unidirectional hypothesis.

Item 2. Are the main EMG reflex response outcomes to be measured clearly described in the methods section?
Yes = 1 / No = 0
If the main outcomes are first mentioned in the Results section, the question should be answered no. Only the outcomes of interest were considered to answer this question.

Item 3. Are the characteristics of the participants included in the study clearly described?
Yes = 1 / No = 0
If there were two or more groups, inclusion/exclusion criteria should be given for all groups. A minimum of 3 relevant criteria should be given for each group to attribute a score of 1. Criteria should answer the question [1] if participants experienced or not back pain, [2] for how long they had back pain or for how long they did not have back pain, and [3] if they had any medical condition?

Item 4.1. Are the perturbation protocol clearly described?
Yes = 1 / No = 0

Item 4.2. Are the protocol of either muscle fatigue, or spinal creep or experimental pain clearly described?
Yes = 1 / No = 0

Item 6. Are the main EMG reflex response findings of the study clearly described?
Yes = 1 / No = 0

Item 7. Does the study provide estimates of the random variability in the data for the main outcomes?
Yes = 1 / No = 0
In non-normally distributed data the inter-quartile range of results should be reported. In normally distributed data the standard error, standard deviation or confidence intervals should be reported. If the distribution of the data is not described, it must be assumed that the estimates used were appropriate and the question should be answered yes.

Item 10. Have actual probability values been reported (e.g. 0.035 rather than <0.05) for the main outcomes except where the probability value is less than 0.001?
Yes = 1 / No = 0

Item 12. Were those subjects who participated representative of the entire population from which they were recruited?
Yes = 1 / No = 0 / Unable to determine = 0

The study must identify the source population for patients. Where a study does not report the proportion of the source population from which the patients are derived, the question should be answered as unable to determine.

Item 16. If any of the results of the study were based on "data dredging", was this made clear?
Yes = 1 / No = 0 / Unable to determine = 0
Any analyses that had not been planned at the outset of the study should be clearly indicated. If no retrospective unplanned subgroup analyses were reported, then answer yes.

Item 18. Were the statistical tests used to assess the main outcomes appropriate?
Yes = 1 / No = 0
The statistical techniques used must be appropriate to the data. If the distribution of the data (normal or not) is not described it must be assumed that the estimates used were appropriate and the question should be answered yes. Statistical analysis plan should correspond to the proposed experiment. When multiple comparison are done, post hoc or planned comparison must be present. The use of covariable must be justified.

Appendix C

Authors years	Subjects & conditions	Perturbation description	EMG	Outcomes	Main findings
Akbari et al. 2015	- 20 cLBP (7M/13F)<br>- 20 healthy (10M/10F)	Standing position: 1% of body mass released from eye level	ES<br>IO<br>EO	- Reflex latency (ms)	- Reflex latency (mean±SD): no difference between groups for ES healthy (56±41) vs ES LBP (59±32), ratio transversus/IO healthy (86±46) vs ratio transversus/IO LBP (88±52) and EO healthy (111±75) vs EO LBP (69±39)
Bazrgari et al. 2011	- 12 healthy (6M/6F) with creep	Standing position: 5mm anterior–posterior external push (T8)	ES<br>RA<br>EO	- Baseline activity (mV)<br>- Reflex latency (ms)	- Baseline (total across all muscles, mean±SD): no difference between pre- (0.043±0.009) and post-creep (0.044±0.011)<br>- Reflex latency (mean±SD)*: no difference for ES between pre- (60.4±3.4) and post-creep (60.6±3.4)
Dupey-ron et al. 2010	- 10 healthy (M) with fatigue	Semi-sitting position: External push at the upper back (pendulum 50% of the total body mass)	ES<br>EO	- Baseline activity<br>- Onset latency (ms)<br>- Reflex amplitude (%MVC)	- Baseline: no difference pre-post fatigue<br>- Reflex latency (mean±SE): no difference between pre- (ES: 89.6±7.5, EO: 82.8±5.9) and post-fatigue (ES: 70.6±9.5, EO: 80.8±6.0)<br>- Reflex amplitude (mean±SE): higher with fatigue for ES (0.815±0.074 vs 0.965±0.106)
Gao et al. 2014	- 21 cLBP (M)<br>- 21 healthy (M)	Standing position: Sudden imbalance simulating a sudden fall	ES<br>MF<br>EO	- Reflex latency (ms)<br>- Reflex amplitude	- Reflex latency (mean±SD): ES decreased in healthy (182.50±51.02) vs LBP (210.28±48.10), no difference between groups for MF and EO<br>- Reflex amplitude: higher in LBP vs healthy for ES (2.90±1.68 vs. 1.77±0.75) and MF (5.18±7.35 vs. 2.00±1.0)
Granata et al. 2001	- 25 healthy (11M/14F) with fatigue	Standing position: Load of 2.5% of maximal lifting capacity released from 0.5m	ES<br>RA<br>IO<br>EO	- Baseline activity (%MVC)	- Baseline (mean±SD): higher with fatigue for right EO (1.4±0.7 vs 2.1±1.3), right IO (3.8±2.1 vs 5.2±3.7), left IO (5.8±3.2 vs 7.1±5.2), right ES (12.2±5.7 vs 14.3±10.4), left RA (1.3±1.6 vs 1.8±2.7); no difference pre-post fatigue for left EO (3.7±7.1 vs 4.4±7.8), right RA (1.3±1.4 vs 1.7±2.8), left ES (3.7±6.5 vs 15.2±10.1)

Granata et al. 2005	- 18 healthy (9F/9M) with creep	Standing position: External push of 75N (T10)	ES<br>RA	- Baseline activity (%MVC)<br>- Reflex latency (ms)<br>- Reflex amplitude (%/N)<br>- Kinematics (mm/N)	- Baseline (mean±SD): no difference for RA between pre- (0.054±0.13) and post-creep (0.059±0.14)<br>- Reflex latency: no difference for ES between pre- (67.43±12.84) and post-creep (67.08±13.87)<br>- Reflex amplitude (mean±SD): higher with creep for ES but not significant (1.20±0.57 vs 1.37±0.76)<br>- Kinematics(mean±SD): lower with creep (570±95.1 vs 545±80.8)
Granata et al. 2004	- 21 healthy (11M/10F) with fatigue	Standing position: 2.27kg load released from 0.5 and 1m	ES<br>RA<br>IO<br>EO	- Baseline activity (%MVC)<br>- Reflex latency (ms)<br>- Reflex amplitude (%MVC)<br>- Kinematics(°)	- Baseline: higher with fatigue for ES (4.74±2.84 vs 6.17±4.36), RA (13.33±4.06 vs 14.23±4.19) and EO (6.5±4.19 vs 7.33±4.24); no difference for IO between pre- (2.8±2.38) and post-fatigue (3.71±3.55)<br>- Reflex latency: No difference pre-post fatigue for ES (126.6± 54.5 vs 125.1±66.3), RA (114.8±57.9 vs 130.8±82.8), IO (87.1±41.8 vs 78.6±58.1) and EO (98.70±39.8 vs 90.8±55.7)<br>- Reflex amplitude: No difference pre-post fatigue for ES (18.75±9.30 vs 19.85±7.79), RA (13.09±6.98 vs 10.81±5.66), IO (17.08±8.77 vs 20.15±9.72) and EO (18.77±6.83 vs 14.71±8.44)<br>- Kinematic: No difference between pre- (10.21±8.05) and post-fatigue (10.57±6.63)
Grondin et al. 2009	- 15 healthy (F) with fatigue	Standing position: 5kg released load from 2.5cm	ES<br>EO<br>IO	- Baseline activity<br>- Reflex amplitude	- Baseline: higher with fatigue for all muscles<br>- Reflex amplitude: no difference between pre- and post-fatigue
Hender-shot et al. 2011	- 12 healthy (6M/6F) with creep	Standing position: 5-mm anterior–posterior external push (T8)	ES<br>RA<br>EO	- Baseline activity (V)<br>- Reflex latency (ms)	- Baseline (total across all muscles, mean±SD): no difference between pre- (0.44±0.096) and post-creep (0.45±0.095)<br>- Reflex latency (mean±SD)*: no difference for ES between pre- (63.3±4.9) and post-creep (63.4±4.3)
Hermann et al. 2006	- 10 healthy (M) with fatigue	Standing position: External push of 170N (inferior	ES	- Baseline activity<br>- Reflex latency (ms)	- Baseline: no difference between conditions<br>- Reflex latency: no difference between pre- (60±18) and post-fatigue<br>- Reflex amplitude: higher with fatigue (0.65±0.28 vs 0.94±0.37)

		margin of the scapulae)		- Reflex amplitude	
Jacobs et al. 2011	- 24 cLBP (13M/11F)<br>- 21 healthy (8M/13F)	Standing position: Linear surface translations (moveable platform)	ES<br>RA<br>IO<br>EO	- Baseline activity<br>- Reflex amplitude	- Baseline: higher for ES and RA muscles in LBP group versus healthy group<br>- Reflex amplitude: no difference between groups
Jones, Henry et al. 2012	- 20 recurrent LBP (9M/11F)<br>- 21 healthy (8M/13F)	Standing position: Linear surface translations (moveable platform)	ES<br>RA<br>IO<br>EO	- Baseline activity<br>- Reflex amplitude	- Baseline: higher in LBP group versus healthy group<br>- Reflex amplitude: higher in LBP group for all trunk muscles (except the right IO) vs healthy participants
Jones, Hitt et al. 2012	- 16 acute LBP (8M/8F)<br>- 16 healthy (8M/8F)	Standing position: Linear surface translations (moveable platform)	ES<br>RA<br>IO<br>EO	- Baseline activity<br>- Reflex amplitude	- Baseline: lower in LBP group for the right EO<br>- Reflex amplitude: higher in LBP group for the left ES and left EO muscles vs healthy participants
Larivière et al. 2010	- 30 cLBP (14M/13F)<br>- 30 healthy (15M/14F)	Semi-sitting position: 50% of the L5/SI extension moment (upper back)	ES<br>RA<br>OE	- Baseline activity (%EMGmax)<br>- Reflex latency (ms)<br>- Reflex amplitude<br>- Kinematics (°)	- Baseline (median (interquartile range 25th and 75th percentiles)): higher in LBP group for ES (for M: 15 (12, 21), for F: 24 (15, 27)) vs healthy participants (for M: 11 (9, 14), for F: 15 (6, 21)). No difference for RA (LBP group for M: 20 (10, 28), for F: 24 (11, 35)); healthy group for M: 18 (10, 38), for F: 31 (15, 35)) and OE (LBP group for M: 15 (8, 28), for F: 28 (18, 48); healthy group for M: 19 (10, 34), for F: 24 (16, 36))<br>- Reflex latency (mean±SD)*: no difference between LBP and healthy participants for ES (at L3 level for F: 92.07±24 vs 97.8±26.4; for M: 89.6±31.1 vs 85.03±15.5)<br>- Reflex amplitude: higher in LBP group for ES vs healthy participants<br>- Kinematics: no difference between groups

Liebe-trau et al. 2013	- 17 cLBP (F)<br>- 17 healthy (F)	Standing position: Sudden loading of 150N at the hand level (hand-held grip)	ES<br>MF<br>RA<br>IO<br>EO	- Baseline activity (μV)<br>- Reflex latency (ms)<br>- Reflex amplitude (μV)	- Baseline (mean±SD): no difference between LBP and healthy groups for ES (2±2 vs 3±2), MF (3±3 vs 2±2), RA (4±3 vs 2±1), IO (14±13 vs 14±11) and EO (9±8 vs 6±3)<br>- Reflex latency (mean±SD): longer in LBP group vs healthy gourp for RA (54±11 vs 39±11) and IO (76±26 vs 55±11). No difference between LBP and healthy participants for ES (74±39 vs 57±31), MF (85±37 vs 79±43) and EO (39±11 vs 33±6)<br>- Reflex amplitude (mean±SD): no difference between LBP and healthy participants for ES (26±24 vs 30±29), MF (27±25 vs 29±34), RA (42±36 vs 65±53), IO (79±71 vs 85±43) and EO (88±53 vs 104±41)
MacDo-nald et al. 2010	- 13 recurrent unilateral LBP (6M/7F)<br>- 14 healthy (8M/6F)	Standing position: 1kg mass released from the eye level	MF	- Baseline activity (μV)<br>- Reflex amplitude (μV)	- Baseline: no difference between groups<br>- Reflex amplitude: lower in LBP group for deep and superficial MF vs healthy group
Mawston et al. 2007	- Group1: 18 healthy (M) with fatigue<br>- Group2: 13 healthy (M) without fatigue	Standing position: Downward vertical force of 100N at a box hold by participants	ES<br>RA<br>IO<br>EO	- Baseline activity<br>- Reflex latency<br>- Kinematics	- Baseline and kinematics: no difference between groups<br>- Reflex latency: shorter in both the control and fatigue groups following exercise for ES and EO
Muslim et al. 2013	- 12 healthy (6M/6F) with creep	Standing position: 5mm anterior–posterior external push (T8)	ES<br>RA<br>EO	- Baseline activity (mV)<br>- Reflex latency	- Baseline (total across all muscles, mean±SD): no difference between pre- (0.054±0.009) and post-creep (0.053±0.01)<br>- Reflex latency (mean±SD): shorter after creep for ES (62.9±4.6 vs 61.3±4)
Newco-mer et al. 2002	- 20 cLBP (9M/11F)<br>- 20 healthy (9M/11F)	Standing position: Footplate perturbation (forward,	ES<br>RA	- Reflex latency (ms)	- Reflex latency (mean±SD)*: no difference between healthy (131±28.1) and LBP participants (138.1±23.6)

		backward and tilting)			
Olson 2014	- 18 healthy (9M/9F) with creep (active and passive conditions)	Standing position: 9.07kg mass released from 1m	ES<br>RA<br>EO	- Baseline activity<br>- Reflex latency<br>- Reflex amplitude<br>- Kinematics (°)	- Baseline: no difference between pre- (ES: 0.042±0.048, RA: 0.060±0.055 and EO: 0.033±0.063) and post-creep<br>- Onset latency: no difference between conditions<br>- Reflex amplitude (mean±SD): no difference pre- (ES: 0.35±0.3, RA: 0.25±0.3, EO: 0.16±0.1) and post-active creep (ES: 0.28±0.3, RA: 0.32±0.4, EO: 0.21±0.3). No difference between pre- (ES: 0.37±0.2, RA: 0.19±0.3, EO: 0.17±0.2) and post-passive creep (ES: 0.38±0.4, RA: 0.25±0.4, EO: 0.15±0.1)<br>- Kinematics (mean±SD): no difference between pre- and post-creep (10.5±5.2 vs 9.1±5.3 for the active condition, and 10.2±5.0 vs 8.6±5.5 for the passive condition)
Rade-bold et al. 2000	- 17 cLBP (12M/5F)<br>- 17 healthy (12M/5F)	Semi-sitting position: Sudden released corresponding to 20%-30% MVC (T9)	ES	- Reflex latency (ms)	- Reflex latency (mean±SD): longer in LBP group for ES (85±25) vs healthy participants (69±8)
Rade-bold et al. 2001	- 16 cLBP (15M/1F)<br>- 14 healthy (13M/1F)	Same as Radebold et al. 2000			- Reflex latency (mean±SD): longer in LBP group for ES (80±20) vs healthy participants (63±9)
Rampra-sad et al. 2010	- 25 cLBP (18M/7F)<br>- 25 healthy (15M/10F)	Standing position: 3kg mass released from 8cm	ES<br>RA	- Reflex latency (ms)<br>- Reflex amplitude	- Reflex latency (mean±SD): longer in LBP group for RA (52±27) and ES (59±22) muscles vs healthy group for RA (53±19) and ES (53±12)<br>- Reflex amplitude: lower in LBP group for RA and ES muscles vs healthy participants
Reeves et al. 2005	- 35 cLBP (10F/27M)<br>- 32 healthy	Semi-sitting position: Sudden released	ES	- Reflex latency (ms)	- Reflex latency (mean±SD): longer in LBP group for ES (82±15) vs healthy participants (62±10)

	(10F/22M)	corresponding to 20% MVC (T9)			
Rogers et al. 2006	- 25 healthy (12M/13F) with creep	Semi-sitting position: External push 75N (T10)	ES RA IO EO	- Baseline activity<br>- Reflex gain (%MVC/N)<br>- Kinematics (mm/N)	- Baseline: lower after creep for EO<br>- Reflex gain (mean±SD): lower after creep for ES (5.06±3.64 vs 3.98±2.46)<br>- Kinematics (mean±SD): no difference between pre- (0.589±0.097) and post-creep (0.539±0.093)
Shan-chez-Zuriaga et al. 2010	- 15 healthy (7M/8F) with fatigue and creep	Standing position: Moving the trunk from 20° to 40° of trunk flexion at 100°/sec	ES	- Reflex latency (ms)<br>- Reflex amplitude (μV)	- Reflex latency (mean±SD): longer after creep for ES (left ES: 60±14, right ES: 65±13) vs pre-creep (left ES: 100±28, right ES: 106±31); no difference between pre- (left ES: 62±15, right ES: 70±20) and post-fatigue (left ES: 71±31, right ES: 76±28)<br>- Reflex amplitude (mean±SD): No difference between pre- (left ES: 176±139, right ES: 218±137) and post-creep (left ES: 176±125, right ES: 155±74); no difference between pre- (left ES: 154±82, right ES: 164±75) and post-fatigue (left ES: 145±73, right ES: 158±84)
Shenoy et al. 2013	- 24 cLBP (16M/8F)<br>- 25 healthy (17M/8F)	Standing position: 3kg mass released from 8cm	ES, RA	- Reflex latency<br>- Reflex amplitude	- Reflex latency: longer in LBP group for RA and ES vs healthy participants<br>- Reflex amplitude (mean): lower in LBP group for RA (24.12 ± 7.18) vs healthy (59.96±17.43) and for ES in LBP (56.89±16.58) vs healthy (83.12±14.28)
Stokes et al. 2006	- 21 acute episodic LBP (10F/11M)<br>- 23 healthy (8F/15M)	Standing position: External push of 5% or 10% of MVC in 80ms (upper back)	ES RA IO EO	- Baseline activity	- Baseline: higher in LBP group for all muscles vs healthy participants
Tooshiza-deh et al. 2013	- 12 healthy (6M/6F) with creep	Standing position: 5 mm anterior–posterior external push (T8)	ES	- Reflex latency	- Reflex latency (mean±SD)*: longer for ES after creep (61.5±5.08 vs 62.5±5.87)

EMG: Electromyography; F: Female; M: Male; LBP: Low back pain; cLBP: Chronic low back pain; MVC: Maximal voluntary contraction; SD: Standard deviation; SE: Standard error; ES: Erector spinae; MF: Multifidus; RA: Rectus abdominis; IO: Internal obliquus; EO: External obliquus.
**Reflex latency values from these articles have been obtained by directly asking the authors.*
